# Fetal intracranial hemorrhage and maternal vitamin K deficiency induced by total parenteral nutrition: A case report

**DOI:** 10.1097/MD.0000000000028434

**Published:** 2022-01-07

**Authors:** Subeen Lee, Hyun Mi Kim, Juyeon Kang, Won Joon Seong, Mi Ju Kim

**Affiliations:** Department of Obstetrics and Gynecology, Kyungpook National University Hospital, School of Medicine, Kyungpook National University, Daegu, Republic of Korea.

**Keywords:** bowel obstruction, early vitamin K supplementation, fetal brain hemorrhage, total parenteral nutrition, vitamin K deficiency

## Abstract

**Rationale:**

Fetal brain hemorrhage is rare. It is caused mainly by maternal trauma or fetal coagulation disorder, but in some cases, vitamin K deficiency may be the cause.

**Patient concerns:**

We describe the case of a pregnant woman with bowel obstruction who was susceptible to vitamin K deficiency due to oral diet restriction, decreased intestinal absorption, and limited intravenous vitamin K supplementation.

**Diagnosis:**

After 18 days of intermittent total parenteral nutrition, acute onset of severe fetal brain hemorrhage developed.

**Interventions:**

After acute onset of fetal brain hemorrhage, the patient underwent an emergency cesarean section at 25 + 3 weeks of gestation due to fetal non-reassuring fetal monitoring.

**Outcomes:**

The Apgar score at birth was 0/0, and despite cardiopulmonary resuscitation, neonatal death was confirmed. After the baby was delivered, we checked the maternal upper abdominal cavity and found a massive adhesion in the small bowel to the abdominal wall near the liver and stomach with an adhesion band. The adhesion band, presumably a complication of previous hepatobiliary surgery, appeared to have caused small bowel obstruction. Adhesiolysis between the small bowel and abdominal wall was performed.

**Lessons:**

This case demonstrates that even relatively short-term total parenteral nutrition can cause severe fetal brain hemorrhage. Vitamin K supplementation is required for mothers who are expected to be vitamin K deficient, especially if they are on total parenteral nutrition for more than 3 weeks.

## 1 Introduction

Fetal brain hemorrhage during pregnancy has a very poor prognosis, and approximately 40% of cases of hemorrhage have been reported to cause fetal death in utero or neonatal death. Otherwise, it results in neurodevelopmental disabilities in more than half of surviving children.^[1]^ Fetal brain hemorrhage occurs in approximately 0.01% of pregnancies.^[1,2]^ The major causes of fetal brain hemorrhage are maternal trauma history or fetal coagulation disorder, but unknown causes have also been reported in about half of all cases. In addition, intrauterine hypoxia and fetal anemia caused by Rh alloimmunization or maternal vitamin K deficiency have been reported to cause fetal brain hemorrhage.^[1]^

Only one-third of the vitamin K concentration in the mother's blood is transferred to the fetus via the umbilical cord, which increases the possibility of fetal brain hemorrhage if the mother takes medication that is involved in vitamin K metabolism or has a persistent malabsorptive condition that causes vitamin K deficiency. Vitamin K supplementation is recommended by the time of delivery if mothers suspected of vitamin K deficiency are at more than 36 weeks of gestation or present with malabsorption for more than 3 weeks.^[3]^

Here, we report the case of a 34-year-old mother who previously had no health-specific problems, such as coagulation disorder, was hospitalized with bowel obstruction and had acute onset fetal intracranial hemorrhage during a relatively short, 2-week total parenteral nutrition (TPN).

## 2 Ethics approval

This study was approved by the Institutional Review Board of Kyungpook National University Chilgok Hospital (IRB No: KNUCH 2021-06-009). Written informed consent was obtained from the patient for publication.

## 3 Case presentation

A 34-year-old pregnant woman (gravida 1, para 0) visited our emergency room at 22 + 5 weeks of gestation because of severe abdominal pain, distension, nausea, and vomiting. The pain was squeezing and repetitive in nature, especially in the left upper quadrant of the abdomen. Her medical history was unremarkable except for a biliary tract operation due to bile duct dilatation during childhood.

The results of routine blood tests, urinalysis, electrocardiography, and chest radiography were unremarkable. Fetal ultrasonography revealed that the fetus was transverse lying with a normal growth pattern and normal amniotic fluid index. Transvaginal ultrasonography revealed a cervical length of 4.73 cm indicating no preterm labor. Abdominal ultrasonography (small bowel and colon) showed a markedly dilated small bowel with increased peristaltic movement and minimal small bowel wall dilatation but no colon dilatation, suggesting small bowel obstruction. Abdominal radiography confirmed a mild paralytic ileus of the small bowel with abundant food material in the stomach. Therefore, she was started on a nil per os diet with intravenous drip infusion (5% dextrose and NaCl). On the 3rd day of admission (23 + 0 weeks of gestation), abdominal pain presented intermittently throughout the day; thus, the patient underwent Gomco suction through a Levin tube to prevent her from ingesting orally. For nutritional and calorie support, she was started on intravenous TPN solution (glucose, amino acids, lipids, and electrolytes) in addition to 5% dextrose fluid. On the 4th day of admission (PA23 + 1 week), vitamins A, B1, B2, B3, B6, C, D, and E were administered intravenously. On the 8th day of admission (23 + 5 weeks of gestation), abdominal radiography showed a focal small bowel ileus. She carefully attempted oral water intake, but failed because of aggravated abdominal distention that ensued. The nil per os diet was resumed.

**Figure 1:**
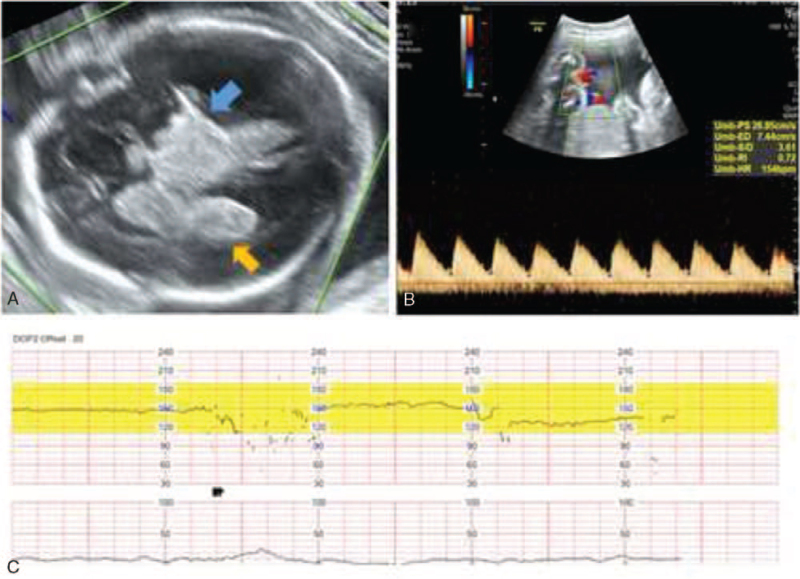
At the diagnosis of fetal brain hemorrhage. (A) Ultrasonography of fetal brain showing high echogenicity in the intracranial space, suspicious of fetal intracranial hemorrhage. Yellow arrow indicates intraventricular hemorrhage, blue arrow indicates thalamic hemorrhage. (B) Umbilical artery velocity wave S/D ratio was 3.2 to 4.0. (C) Nonstress test (NST) showed deceleration of fetal heart rate.

**Figure 2:**
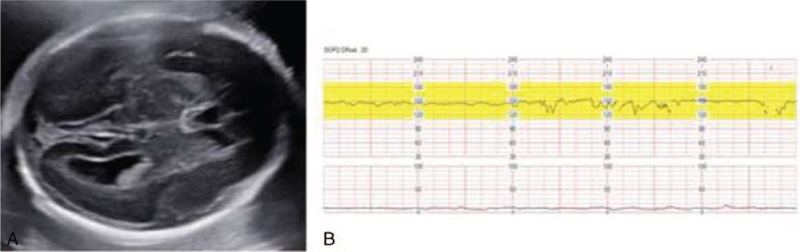
Data from examinations the day before fetal brain hemorrhage. (A) Ultrasonography of fetus conducted the day before fetal brain hemorrhage was diagnosed. No signs of fetal brain abnormalities including brain hemorrhage were evident. (B) Nonstress test (NST) showed moderate to good variability. The mother did not complain about decreased fetal movement.

**Figure 3:**
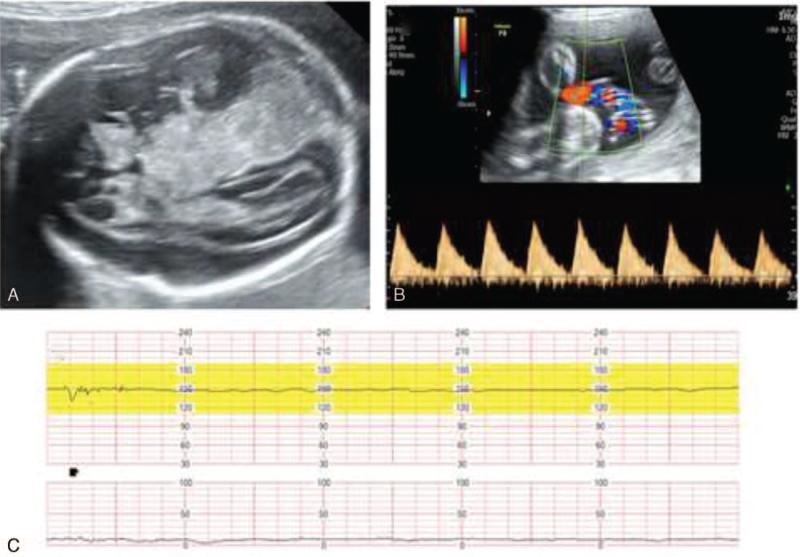
Six hours after diagnosis of fetal brain hemorrhage. (A) Ultrasonography of fetal brain. Massive fetal intracranial hemorrhage was observed. (B) Doppler ultrasonography of umbilical artery, indicating absent diastolic flow. (C) Nonstress test (NST) showing minimal fetal variability.

On day 18 (25 + 3 weeks of gestation), she complained of decreased fetal movement. The nonstress test (NST) showed minimal to moderate fetal variability. On ultrasonography, the fetal intracranial space showed high echogenicity, suspicious of fetal intracranial hemorrhage, and umbilical artery velocity wave S/D ratio was 3.2 to 4.0 (Fig. 1) compared with a previous scan (Fig. 2). In the follow-up NST after 2 hours, fetal variability was minimal, and on serial ultrasonographic examination, fetal brain lesions suspicious of intracranial hemorrhage increased. Doppler ultrasonography of the umbilical artery indicated an absence of diastolic flow (Fig. 3). All signs indicated an impending fetal demise. Fetal salvage was attempted via emergency cesarean section. The Apgar score at birth was 0/0, and despite cardiopulmonary resuscitation, neonatal death was confirmed. After the baby was delivered, we checked the maternal upper abdominal cavity and found a massive adhesion in the small bowel to the abdominal wall near the liver and stomach with an adhesion band. The adhesion band, presumably a complication of previous hepatobiliary surgery, appeared to have caused small bowel obstruction. Adhesiolysis between the small bowel and abdominal wall was performed with the assistance of the general surgery department.

## 4 Discussion

Fetal brain hemorrhage occurs in approximately 1 in 10,000 pregnancies, most commonly in the third trimester of pregnancy, arising from germinal matrix hemorrhage.^[1,4]^ It is devastating for both parents and fetuses, either with intrauterine death or severe neurodevelopmental complications in surviving children.^[1,2]^ According to previous reports, the causes of fetal brain hemorrhage may be trauma, coagulopathy, infection, drug use, febrile illness, and cholestasis of pregnancy as maternal factors, and thrombocytopenia, congenital factor X, factor V deficiency, vascular malformations, and monochorionic twin complications as fetal factors.^[4]^ In addition, placenta and umbilical problems, or vitamin K deficiency, can also cause fetal hemorrhage.

Vitamin K is a cofactor that activates other coagulation factors, such as II, VII, IX, X, protein C, and protein S^[5]^; thus, its deficiency is a well-known cause of coagulopathy.^[6]^ Vitamin K is stored within the body for short periods and is easily eliminated.^[7]^ Therefore, vitamin K should be supplied regularly through oral intake or produced by the gastrointestinal microbiome in the large intestine.^[8]^ Although vitamin K deficiency is rare among the population with normal oral intake, it can be induced due to poor oral intake (such as in hyperemesis gravidarum), gastrointestinal diseases that prevent vitamin K absorption, or liver diseases.^[5]^

During pregnancy, only one-third of the vitamin K in the mother's blood is transferred to the fetus,^[9]^ which increases the possibility of fetal brain hemorrhage if the mother takes certain medications that are involved in vitamin K metabolism or has a malabsorptive condition. Previous studies have reported that diseases such as hyperemesis gravidarum, eating disorders, and Crohn disease cause vitamin K deficiency during pregnancy, resulting in fetal brain hemorrhage.^[2,3,10,11]^ In most cases, a relatively prolonged period of an eating disorder or intestinal absorptive disorder caused vitamin K deficiency, and recommendations included vitamin K supplementation for pregnant women with more than 3 weeks of TPN or more than 36 weeks of pregnancy impending delivery.^[3]^

In the present case, the patient developed an intestinal obstruction during the second trimester of pregnancy, leading to sudden and fatal brain hemorrhage of the fetus in the absence of maternal bleeding tendency. In contrast to a previous report, fetal hemorrhage occurred within a relatively short period of 18 days of TPN, which was likely caused not only by vitamin K deficiency in oral intake or TPN, but also by decreased vitamin K intestinal absorption and changes in intestinal normal flora. Therefore, vitamin K supplementation must be considered from a relatively early date, considering that even a short TPN period may present a combination of factors that can cause vitamin K deficiency.

In previous reports, deliveries were performed at least a few days or weeks after confirming fetal intracranial hemorrhage, and usually in the third trimester of pregnancy.^[1–3,11]^ In this case, the patient complained of decreased fetal movement and immediate diagnosis of fetal intracranial hemorrhage through abdominal ultrasonography, and decreased fetal variability was observed through NST. In serial monitoring, the amount of hemorrhage in the fetus increased rapidly in a matter of hours, and the pattern of fetal variability in NST showed unguaranteed fetal well-being and absent diastolic flow of umbilical artery Doppler findings. Therefore, emergency cesarean section was performed 6 hours after diagnosis of fetal brain hemorrhage. This is thought to have caused hemorrhage and progressed rapidly as the fetus’ brain was vulnerable at an early gestational age.

## 5 Conclusion

Healthy mothers without any coagulation disorder placed on a relatively short period of TPN can develop vitamin K deficiency. Vitamin K supplementation must be considered from a relatively early date, especially if the mother is suspected of malabsorption, such as intestinal obstruction. In addition, regardless of the gestational age, the mother must be considered a high-risk patient with a high chance of fetal hemorrhage. Furthermore, the possibility of fetal hemorrhage must be considered, and early diagnosis must be made through ultrasound in the event of decreased fetal movement.

## Author contributions

**Conceptualization:** Won Joon Seong.

**Data curation:** Juyeon Kang.

**Supervision:** Mi Ju Kim.

**Writing – original draft:** Subeen Lee, Hyun Mi Kim.

**Writing – review & editing:** Mi Ju Kim.

## References

[1] Ghi T Simonazzi G Perolo A. Outcome of antenatally diagnosed intracranial hemorrhage: case series and review of the literature. Ultrasound Obstet Gynecol 2003;22:121–30. 12905503 10.1002/uog.191

[2] Abu-Rmaileh M Ramseyer AM Burdine L Dajani NK. Fetal intracranial hemorrhage associated with maternal coagulopathy and vitamin K deficiency after biliary drain placement: a case report and literature review. Case Rep Womens Health 2021;31:e00329. 34041000 10.1016/j.crwh.2021.e00329 PMC8144653

[3] Sotodate G Matsumoto A Konishi Y Toya Y Endo M Oyama K. Fetal intracranial hemorrhage due to maternal subclinical vitamin K deficiency associated with long-term eating disorder. J Obstet Gynaecol Res 2019;45:461–5. 30255653 10.1111/jog.13825

[4] Putbrese B Kennedy A. Findings and differential diagnosis of fetal intracranial haemorrhage and fetal ischaemic brain injury: what is the role of fetal MRI? Br J Radiol 2017;90:20160253. 27734711 10.1259/bjr.20160253 PMC5685119

[5] Mijares ME Nagy E Guerrero B Arocha-Piñango CL. Vitamin K: biochemistry, function, and deficiency. Review. Invest Clin 1998;39:213–29. 9780555

[6] Alperin JB. Coagulopathy caused by vitamin K deficiency in critically ill, hospitalized patients. Jama 1987;258:1916–9. 3656602

[7] Bultynck C Munim N Harrington DJ. Prevalence of vitamin K deficiency in older people with hip fracture. Acta Clin Belg 2020;75:136–40. 30618350 10.1080/17843286.2018.1564174

[8] Booth SL Suttie JW. Dietary intake and adequacy of vitamin K. J Nutr 1998;128:785–8. 9566982 10.1093/jn/128.5.785

[9] Suzuki S Iwata G Sutor AH. Vitamin K deficiency during the perinatal and infantile period. Semin Thromb Hemost 2001;27:93–8. 11372776 10.1055/s-2001-14066

[10] Kawamura Y Kawamata K Shinya M Higashi M Niiro M Douchi T. Vitamin K deficiency in hyperemesis gravidarum as a potential cause of fetal intracranial hemorrhage and hydrocephalus. Prenat Diagn 2008;28:59–61. 18058978 10.1002/pd.1903

[11] Hirose M Akiyama M Takakura K Noda Y. Active Crohn disease with maternal vitamin K deficiency and fetal subdural hematoma. Obstet Gynecol 2001;98:919–21. 11704203 10.1016/s0029-7844(01)01331-x

